# Contribution of Pyk2 pathway and reactive oxygen species (ROS) to the anti-cancer effects of eicosapentaenoic acid (EPA) in PC3 prostate cancer cells

**DOI:** 10.1186/s12944-019-1122-4

**Published:** 2020-01-31

**Authors:** Keiichi Oono, Kazuo Ohtake, Chie Watanabe, Sachiko Shiba, Takashi Sekiya, Keizo Kasono

**Affiliations:** 1 Laboratory of Physiology, Faculty of Pharmaceutical Sciences, Josai University, 1-1 Keyakidai, Sakado, Saitama, 350-0295 Japan; 2 Laboratory of Clinical Pathology, Faculty of Pharmaceutical Sciences, Josai University, 1-1 Keyakidai, Sakado, Saitama, 350-0295 Japan

**Keywords:** PC3, EPA, ROS, ERK, Pyk2

## Abstract

**Background:**

n-3 polyunsaturated fatty acids (n-3 PUFAs), including eicosapentaenoic acid (EPA) and docosahexaenoic acid (DHA), are thought to exert protective effects in cardiovascular diseases. In addition, n-3 PUFAs have demonstrated anti-cancer effects in vitro and in vivo.

**Objective:**

We investigated the anti-cancer effects and mechanism of action of EPA on PC3 prostate cancer cells in vitro.

**Methods:**

PC3 cells were treated with various concentrations of EPA, and cell survival and the abilities of migration and invasion were evaluated. The time course of the growth inhibitory effect of EPA on PC3 cells was also assessed. The mechanism underlying the anti-cancer effects of EPA was investigated by human phosphokinase and human apoptosis antibody arrays, and confirmed by western blot analysis. We also examined the contribution of reactive oxygen species (ROS) to the effects of EPA using the ROS inhibitor N-acetyl cysteine.

**Results:**

EPA decreased the survival of PC3 cells in a dose-dependent manner within 3 h of application, with an effective concentration of 500 μmol/L. EPA inhibited proline-rich tyrosine kinase (Pyk)2 and extracellular signal-regulated kinase 1/2 phosphorylation as determined by western blotting and the antibody arrays. The growth of PC3 cells was inhibited by EPA, which was dependent on ROS induction, while EPA inhibited Pyk2 phosphorylation independent of ROS production.

**Conclusions:**

Inhibition of Pyk2 phosphorylation and ROS production contribute to the anticancer effects of EPA on PC3 cells.

## Introduction

Prostate cancer (PC) is the second most common cancer in men worldwide, with the incidence increasing in Asian countries, including Japan [1, 2]. Standard treatments for PC include surgery, radiation, hormone therapy, and chemotherapy. Advanced PC with metastasis is treated with androgen deprivation therapy (ADT) in association with medical or surgical castration [3]. However, after several years of treatment, patients ultimately develop castration resistance; non-metastatic or metastatic castration-resistant (CR) PC is refractory to ADT and develops mechanisms to proliferate irrespective of castration. Furthermore, anti-androgen therapy can worsen the patient’s condition by stimulating PC growth. This has been demonstrated with flutamide therapy and is known as anti-androgen withdrawal syndrome [4]. Only a few chemotherapeutics and radiation have been developed for the treatment of CRPC [5], and these have not improved the poor prognosis of this disease [6].

There is growing evidence that n-3 polyunsaturated fatty acids (n-3 PUFAs) found in fish oil—especially eicosapentaenoic acid (EPA) and docosahexaenoic acid (DHA)—can improve lipid metabolism and blood lipid profiles, prevent the progression of atherosclerosis, and reduce the incidence of cardiovascular diseases [7] as well as liver and plasma triglyceride levels [8]. We previously demonstrated that chronic oral administration of EPA prevented endothelial dysfunction in a mouse model of type 2 diabetes [9]. n-3 PUFAs have also been reported to have various anti-cancer effects in several types of malignancy in vitro and in vivo. n-3 PUFAs inhibit extracellular signal-regulated kinase (ERK) and Akt signaling pathways and show anti-cancer effects in breast cancer [10], reduce the incidence of liver cancer in hepatitis virus-infected patients [11], and lower the risk of pancreatic cancer [12]. Cis-unsaturated fatty acids including EPA are easily incorporated into cancer cells and induce free radical generation, inducing tumoricidal action [13–16]. However, other studies have suggested that there is insufficient evidence for a significant association between n-3 PUFAs and cancer incidence [17].

We previously reported that EPA and DHA have anti-proliferative, −migratory, and -invasive effects in the PC3 CRPC cell line [18]. We also observed that the combination of anti-cancer drugs and n-3 PUFAs synergistically inhibited the proliferation of PC3 cells (unpublished data). However, the mechanism of action of n-3 PUFAs, and specifically, the molecular basis for the inhibitory effects of EPA on PC3 cell proliferation, migration, and invasion has yet to be defined.

Various signaling pathways control cell proliferation; their dysregulation can lead to over-proliferation and aberrant migration and invasion. Tyrosine kinase (TK) is an important mediator of cell proliferation. Receptor (R) TK is a transmembrane receptor with TK activity, whereas non-receptor (NR) TK is present in the cytoplasm and comprises Abl, Src, and proline-rich TK (Pyk)2 families. Various NRTKs are expressed in PC3 cells, and Src plays a role in multiple biological processes in PC cells [19]. Activation of these TKs leads to cell proliferation through activation of transcription factors that promote cell cycle progression.

NRTKs enhance the proliferation of cancer cells to promote their survival, so suppressing these pathways can lead to cell growth inhibition or death. Most cancer cells are resistant to apoptosis and activate pathways that suppress the pro-apoptotic active form of caspases [20]. Moderate ROS levels may cause tumorigenesis through DNA damage in pro-tumorigenic cells [21]. Thus, eliminating or reducing ROS production is a potential strategy for cancer therapy, since ROS also regulate the activation of several RTKs and NRTKs [22]. On the other hand, EPA-induced ROS overproduction stimulates apoptotic signals in HepG2 liver cancer cells [23], and oxidative stress causes PC3 cell death by stimulating mitochondrial ROS production and apoptosis [24]. DHA has been found to cause cell death by inducing ROS production in PC3 and DU145 PC cells [25].

In the present study, we investigated the mechanism underlying the anti-cancer effects of n-3 PUFA EPA, first by characterizing the optimal concentration and time required for these effects in PC3 cells, and then by analyzing protein expression by western blotting and antibody arrays. To clarify the effects of ROS production induced by EPA, we used the ROS inhibitor N-acetyl cysteine (NAC).

## Materials and methods

### Reagents

EPA (Sigma-Aldrich, St. Louis, MO, USA) was dissolved in 99% isopropanol (Wako, Tokyo, Japan) and stored at − 30 °C. For experiments, EPA was dissolved in serum-free Dulbecco’s modified Eagle’s medium (DMEM; Gibco, New York, NY, USA) containing 3% bovine serum albumin (BSA) (Sigma-Aldrich), and NAC (Wako) was dissolved in phosphate-buffered saline (PBS). Human phospho-kinase array (R&D Systems, Minnesota, MN, USA) and the human apoptosis antibody Array Membranes (Abcam, Cambridge, UK) were used for protein analysis. Antibodies against ERK1/2, phosphorylated (p)-ERK1/2, Pyk2, p-Pyk2, and β-actin were from Cell Signaling Technology Japan (Tokyo, Japan) and were diluted with Can Get Signal reagent (Toyobo, Osaka, Japan).

### Cell culture

PC3 cells (Riken BRC Cell Bank, Tsukuba, Japan) were grown in DMEM (Gibco) supplemented with 10% heat-inactivated fetal bovine serum (FBS), 100 U/mL of penicillin, and 100 μg/mL of streptomycin (Gibco). The cells were cultured at 37 °C in a humidified 5% CO_2_ atmosphere.

### Cell proliferation assay

PC3 cells were seeded in 35-mm dishes at a density of 3 × 10^5^ cells/dish in DMEM containing 10% FBS and incubated for 24 h, and the culture medium was replaced with serum-free DMEM after washing with PBS. After an additional 24 h, the cells were washed with PBS, and serum-free medium containing 3% BSA and various concentrations of EPA (100–500 μM) were added. After 24 h, the number of cells was counted with a hemocytometer.

### Time course of cell proliferation

PC3 cells were seeded in a 35-mm high μ-Dish Grid-500 (Ibidi, Planegg, Germany) at a density of 3 × 10^5^ cells/dish in DMEM containing 10% FBS. After 24 h, the cells were washed with PBS, and serum-free DMEM was added; after another 24 h, the cells were washed with PBS, and serum-free medium containing 3% BSA with or without EPA (500 μM) was added. Cells were imaged with a microscope and counted at the indicated times.

### Migration and invasion assay

Migration and invasion were assessed as described in our previous study [18]. Briefly, we used Transwell membranes (8-μm pore size; Corning Inc., Corning, NY, USA) with or without Matrigel coating. PC3 cells were cultured in the upper chamber of a Transwell insert in serum-free medium containing various concentrations of EPA. The lower chamber was filled with DMEM containing 10% FBS. For the migration assay, Matrigel-uncoated upper chambers were used; cells were seeded at a density of 2 × 10^5^ cells/mL, and after 24 h, the number of cells that had migrated into the lower chamber was counted. For the invasion assay, Matrigel-coated upper chambers were used. Before seeding the cells, Matrigel was fixed by incubation for 90 min, and cells were seeded at a density of 1 × 10^5^ cells/mL. After 48 h, cells that had invaded into the lower chamber were counted.

### Antibody arrays

PC3 cells were cultured in DMEM containing 10% FBS for 24 h, then cultured in serum-free medium for an additional 24 h before treatment with EPA (500 μM) or vehicle as a control. After 2 h of incubation with EPA or vehicle, samples were prepared for the antibody array according to the manufacturers’ protocols. Chemiluminescence detection was performed on a Lumi Cube (Liponics, Tokyo, Japan) using Clarity Western ECL Substrate (Bio-Rad, Hercules, CA, USA). Images of the arrays were analyzed using Image J software (National Institutes of Health, Bethesda, MD, USA).

### Inhibition of ROS production

PC3 cells were cultured in a 35-mm dish at a density of 3 × 10^5^ cells/dish in DMEM containing 10% FBS for 24 h, and then washed with PBS and incubated in serum-free DMEM for an additional 24 h. The cells were washed with PBS, and EPA (500 μM) with or without 5 mM of NAC (Wako) was added, cells were incubated for 24 h, and cell numbers were counted with a hemocytometer.

### Western blot analysis

PC3 cells were cultured in 60-mm dishes at a density of 8 × 10^5^ cells/dish in 10% FBS-containing DMEM for 24 h. PC3 cells were cultured with serum starvation for an additional 24 h, then treated with EPA (500 μM) or vehicle. The cells were lysed in M-PER (Thermo Fisher Scientific, Waltham, MA, USA) and centrifuged at 13,000 rpm (30,250 g) at 4 °C for 10 min. Protein samples were diluted in 2× Laemmli sample buffer (Bio-Rad), then boiled at 100 °C, and 20 μg of protein were loaded in each lane of a TGX precast gel (Bio-Rad) and resolved by electrophoresis (100 V, 0.3 A). The proteins were transferred to an Immun-Blot polyvinylidene difluoride membrane (Bio-Rad) at 100 V and 0.3 A for 1 h. The membrane was blocked with Block Ace (KAC, Kyoto, Japan) for 1 h and probed with antibody solution for 1 h at room temperature. The membrane was washed with Tris-buffered saline (Bio-Rad) containing 1% Tween 20. Lumi Cube and Clarity Western ECL substrate were used for signal detection, and protein band intensity was analyzed using Image J software.

### Statistical analysis

Statistical analyses were performed using R v.3.4.3 (https://www.r-project.org/). Data are presented as means ± standard error, and differences between means were evaluated with Dunnett’s test. A *P* value less than 0.05 was considered statistically significant.

## Results

### EPA suppresses PC3 cell proliferation

**Fig. 1:**
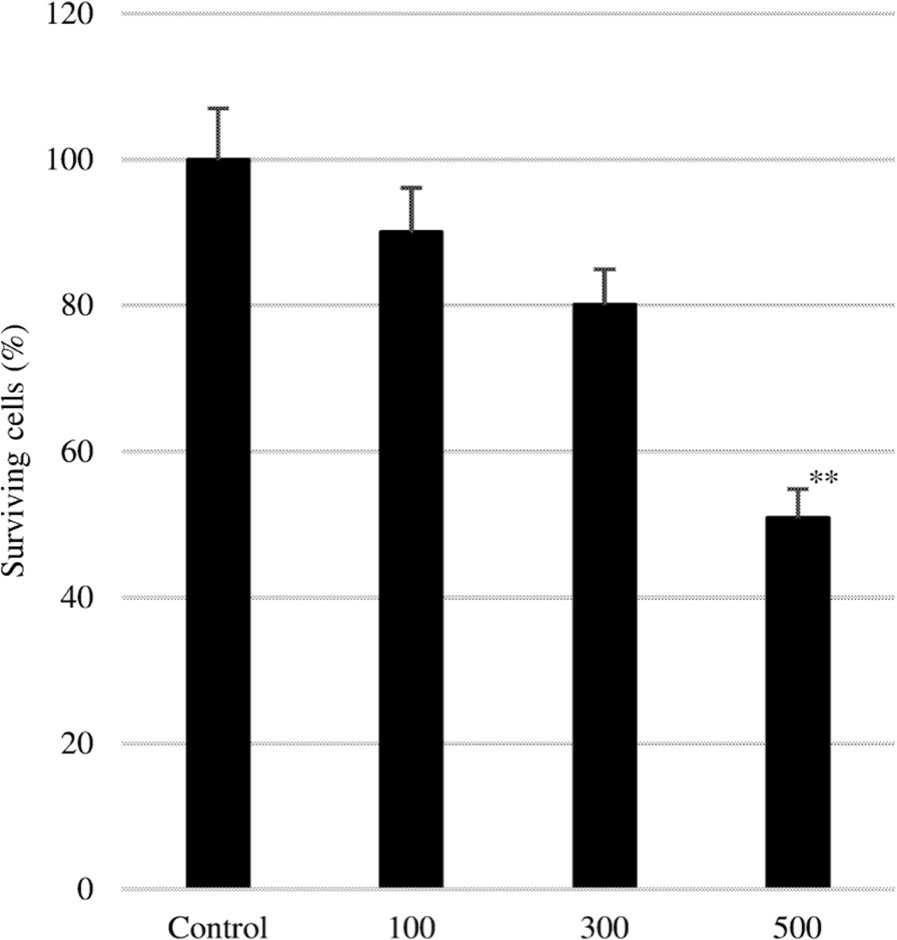
Effect of EPA on PC3 cell proliferation. After 24 h of culture in serum-free medium, various concentrations of EPA (0, 100, 300, and 500 μM) were added to the cultures. Data represent mean + SEM (*n* = 3). **P < 0.01 vs. 0 μM

EPA inhibited the proliferation of PC3 cells in a dose-dependent manner (Fig. 1). However, only the highest concentration of EPA (500 μM) significantly reduced the number of cells to 50% of the control value.

**Fig. 2:**
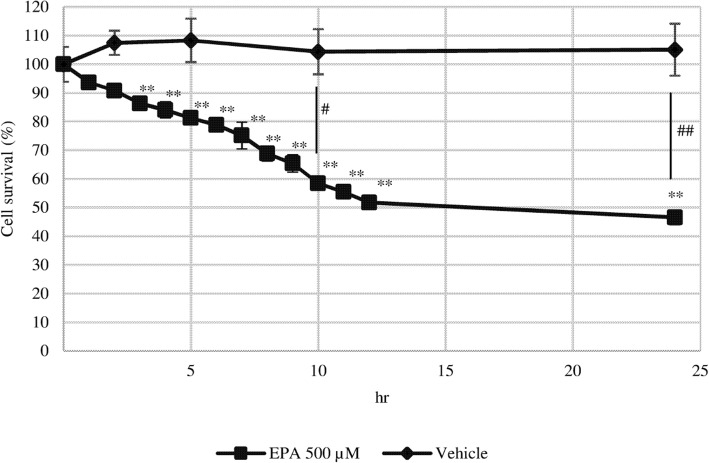
Effect of EPA on the time course of PC3 cell proliferation. After 24 h of culture in serum-free medium, EPA (500 μM), or vehicle was added to the cells. Data represent mean ± SEM (*n* = 3). **P < 0.001 vs 0 h; ^#^*P* < 0.05, ^##^*P* < 0.01 v. vehicle

In serum-free medium, the number of PC3 cells in the control group did not change over a 24-h period, but gradually decreased with EPA treatment, with significant differences detected after 3 h. After 12 h, the number of cells was 50% of the control value and remained constant up to 24 h (Fig. 2).

### EPA inhibits PC3 cell migration and invasion

**Fig. 3:**
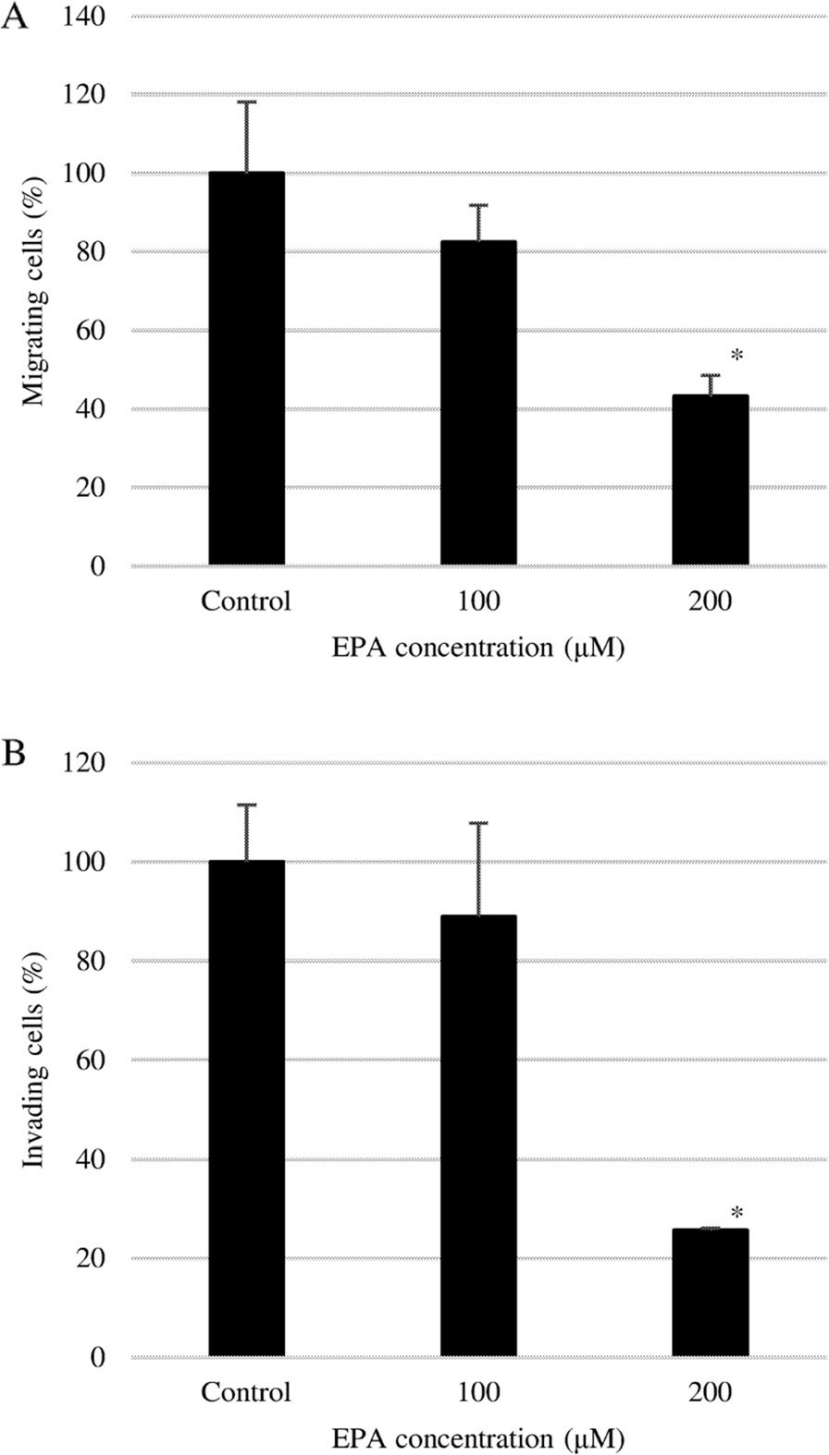
Effect of EPA on EPA on PC3 (**a**) cell migration and (**b**) invasion. Double-chambered cell culture dishes with a transwell insert separating the two chambers were used to evaluate PC3 cell migration and invasion. Cells were seeded in the upper chamber, which was uncoated (migration) or coated (invasion) with Matrigel, while the lower chamber was filled with DMEM containing 10% FBS. Data represent mean + SEM (*n* = 3). *P < 0.05

EPA inhibited both PC3 cell migration and invasion in a dose-dependent manner, and the rate of migration and invasion relative to the control was 43% (Fig. 3a) and 26% (Fig. 3b), respectively, at 200 μmol/L EPA. Thus, PC3 cell invasion was inhibited to a greater extent than migration by EPA.

### Proteins associated with phosphokinase and apoptosis pathways are activated by EPA treatment

**Fig. 4:**
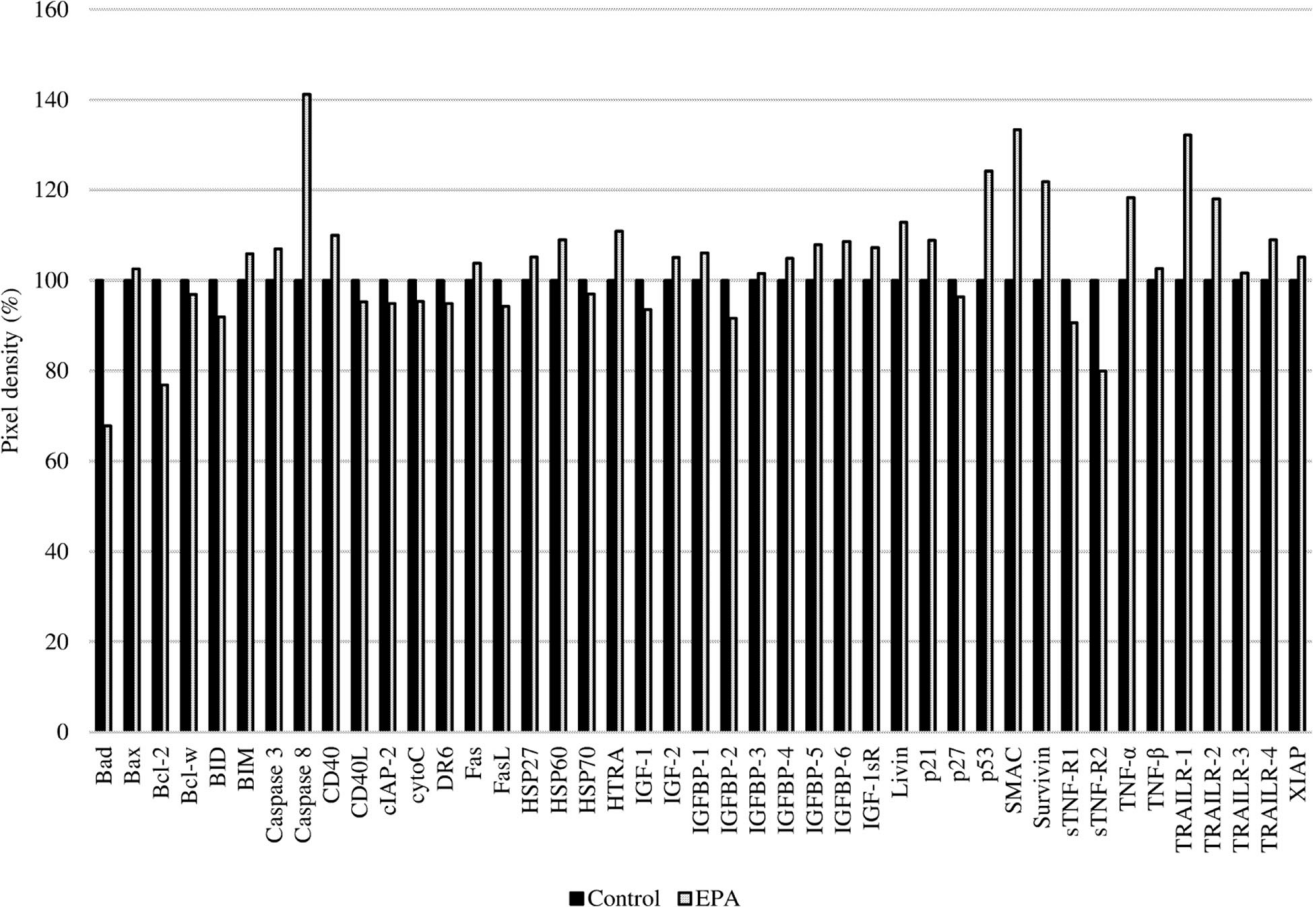
Results of the apoptosis antibody array. PC3 cells with or without EPA treatment were analyzed. The y axis shows the pixel density (%). The expression level of each protein in the control was adjusted to 100%

**Fig. 5:**
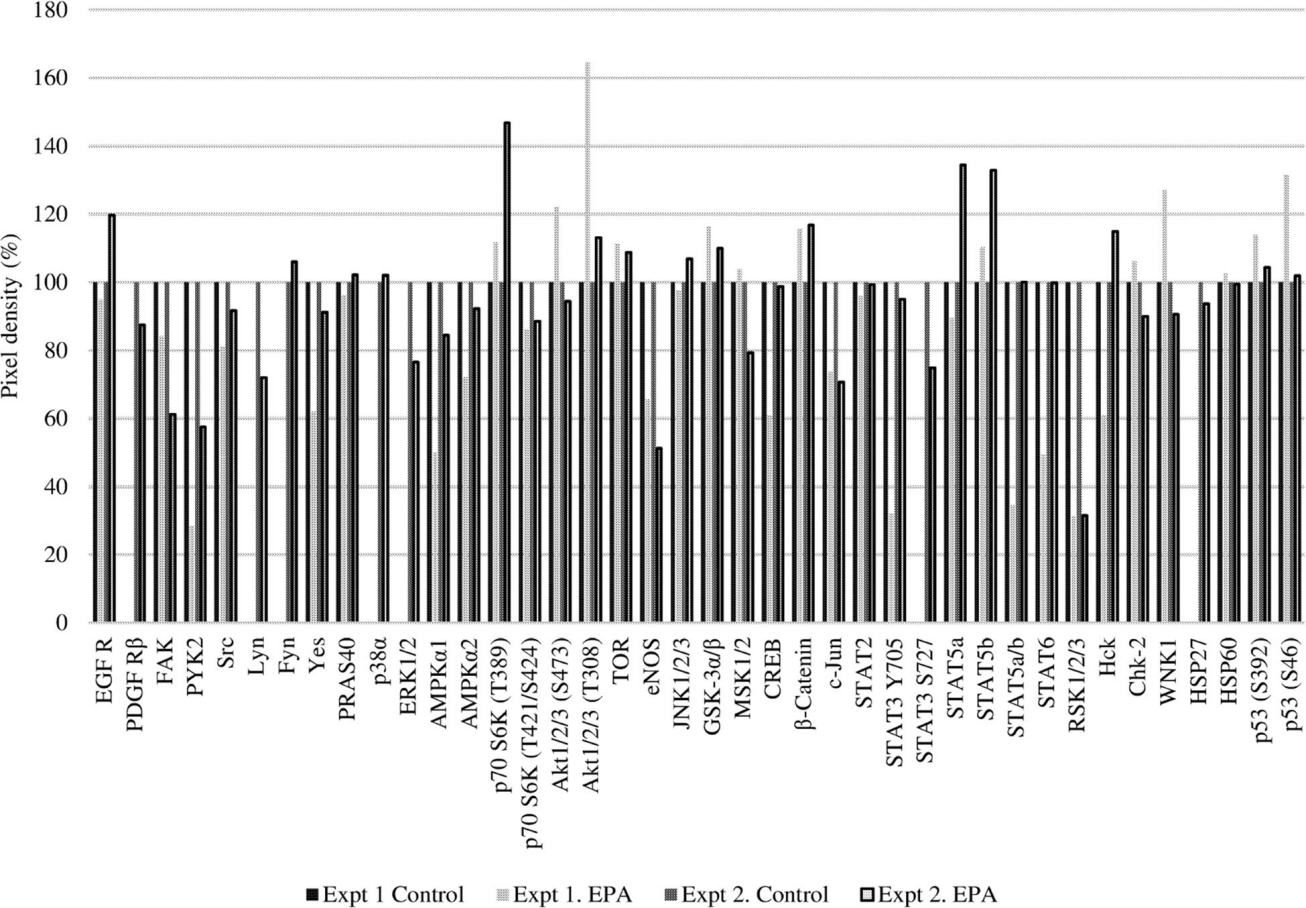
Results of two experiments using the human phosphokinase array. The y axis shows pixel density (%). For the control groups in experiments 1 and 2 (Expt1 Control and Expt2 Control, respectively), the expression level of each protein was adjusted to 100%. For the EPA-treated groups in experiments 1 and 2 (Expt1 EPA and Expt2 EPA, respectively), the expression level of each protein was determined relative to that in the corresponding control group

Using two types of antibody arrays, we determined that proteins involved in the phosphokinase and apoptosis pathways were activated by EPA treatment. Based on the results shown in Fig. 2, protein samples for antibody arrays and western blotting were prepared 2 h after adding 500 μmol/L EPA. More significant results were obtained between the EPA-treated and untreated control groups using the phosphokinase as compared to the apoptosis array (Figs. 4 and 5). The expression of apoptosis-related proteins including soluble tumor necrosis factor receptor (sTNF-R)2, second mitochondria-derived activators of caspase (SMAC), Survivin, tumor necrosis factor related apoptosis-inducing ligand receptor (TRAIL-R)1, caspase 8, B-cell lymphoma (Bcl)-2, and p53 differed by more than 20% between the two groups (Table 1), with sTNF-R2 and Bcl-2 being downregulated and the other proteins being upregulated by EPA treatment.

**Table 1:**
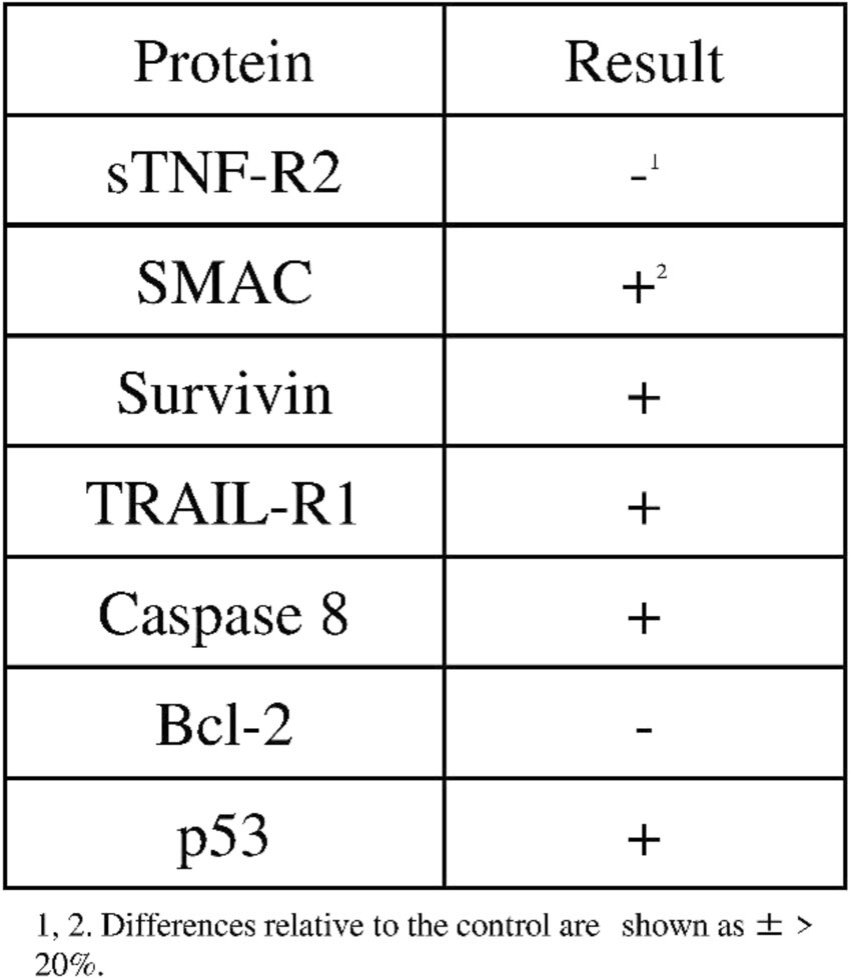
Difference in expression levels of various proteins relative to the control in PC3 cells treated with EPA

1, 2. Differences relative to the control are shown as ± > 20%

The phosphokinase array data showed increased or decreased phosphorylation of several kinases (Fig. 5 and Table 2). EPA reduced the phosphorylation of several proteins by over 20% relative to the control, including Pyk2, endothelial nitric oxide synthase (eNOS), c-Jun, and ribosomal S6 kinases (RSK)1/2/3.

**Table 2:**
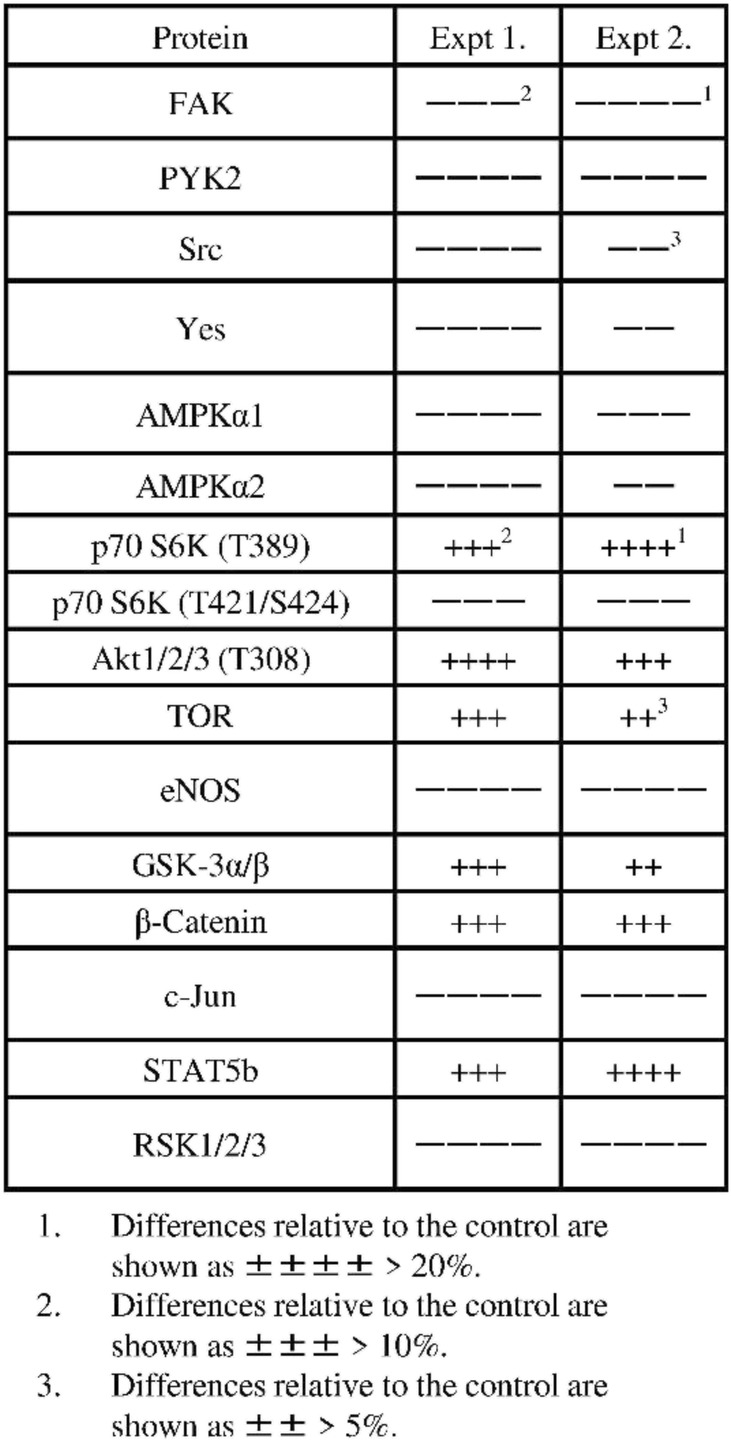
Differences in the expression levels of various proteins in EPA-treated PC3 cells relative to the control (related to Fig. 5)

1. Differences relative to the control are shown as ± ± ± ± > 20%

2. Differences relative to the control are shown as ± ± ± > 10%

3. Differences relative to the control are shown as ± ± > 5%

### Contribution of ROS to PC3 cell death

**Fig. 6:**
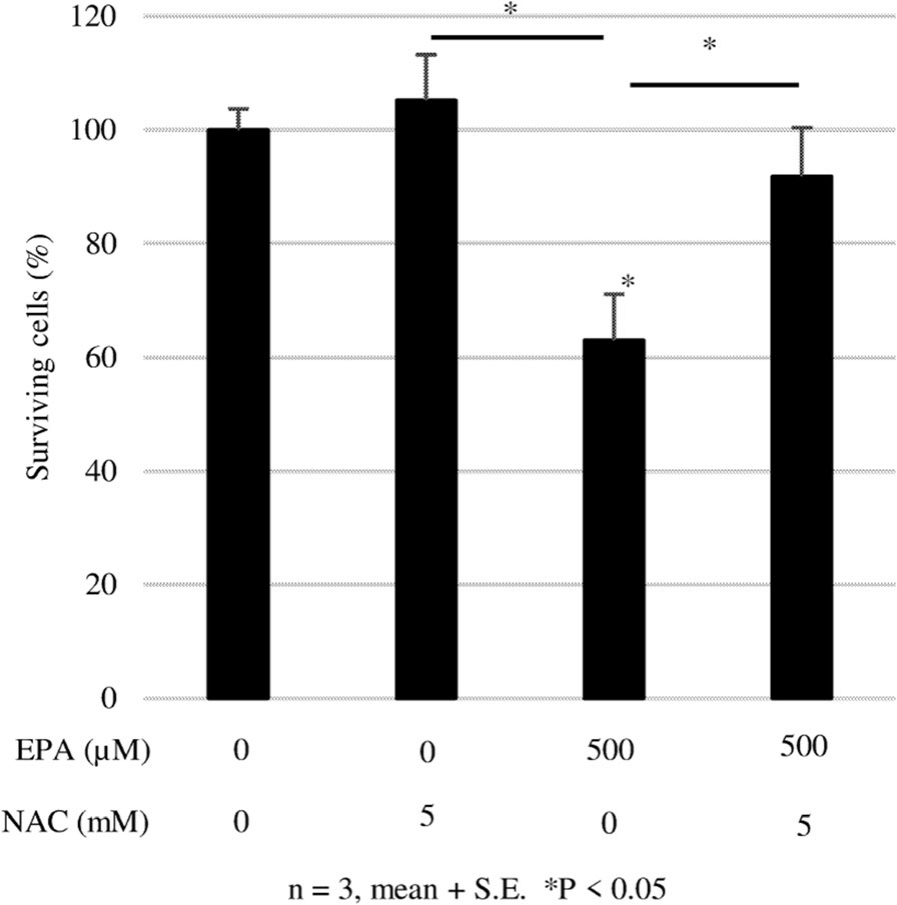
Effect of NAC on the anti-cancer effect of EPA against PC3 cells. After 24 h of culture in serum-free medium, cells were treated with EPA (500 μM) or NAC or both. Data represent mean + SEM (*n* = 3). *P < 0.05

We next analyzed the contribution of ROS to the effects of EPA on PC3 cell survival and found that EPA treatment decreased survival to 60% of the control rate, whereas NAC (5 mmol/L) had no effect. Treatment with both EPA and NAC restored the survival rate to the control level. Thus, NAC abrogates the anti-cancer effect of EPA against PC3 cells (Fig. 6).

### EPA inhibits ERK and Pyk2 phosphorylation

**Fig. 7:**
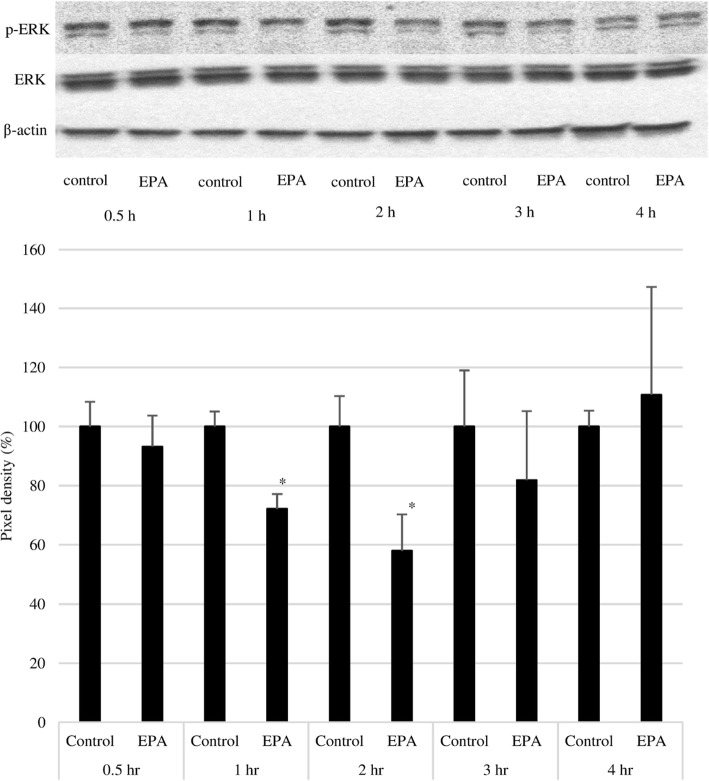
Effect of EPA on ERK1/2 phosphorylation. Upper panel: Representative results of western blot analysis. Lower panel: Pixel density of the control group at the indicated times was adjust to 100%; relative pixel density of EPA-treated PC3 cells is shown. Data represent mean + SEM (*n* = 3). *P < 0.05

**Fig. 8:**
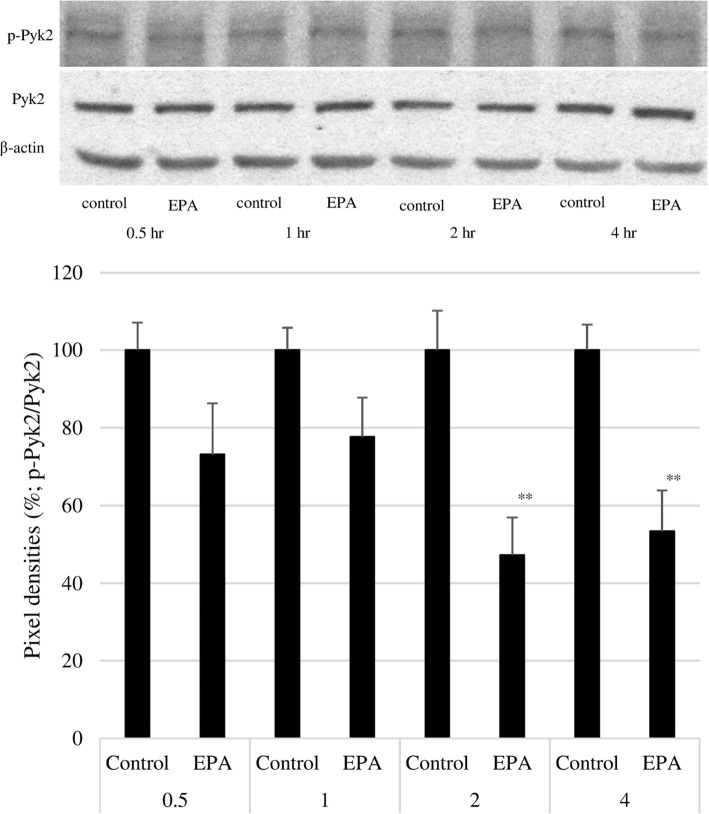
Effect of EPA on Pyk2 phosphorylation. Upper panel: Representative result of western blot analysis. Lower panel: Pixel density of the control group at the indicated times was adjusted to 100%; relative pixel density of EPA-treated PC3 cells is shown. Data represent mean + SEM (*n* = 3). *P < 0.05

**Fig. 9:**
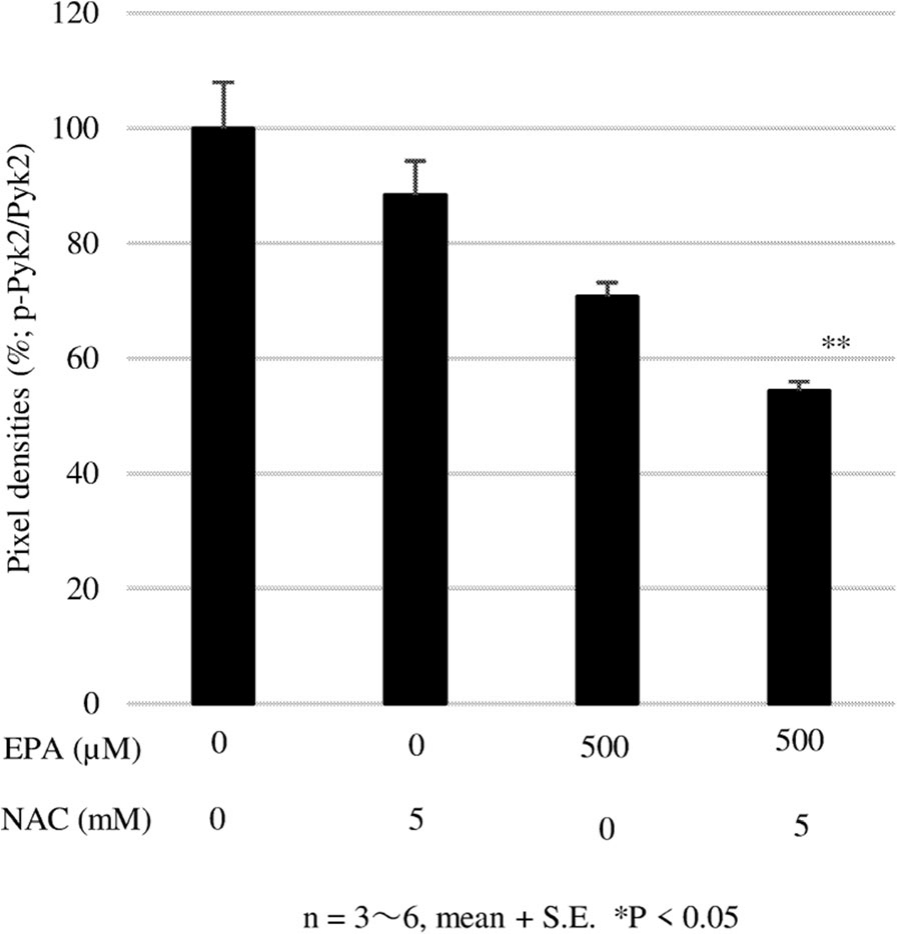
NAC enhances the inhibition effect of EPA on Pyk2 phosphorylation. After 24 h of culture in serum-free mediumm, PC3 cells were treated with EPA (500 μM) or NAC or both. Data represent mean + SEM (n + 3). *P < 005

To confirm the mechanism of action of EPA, cells were treated at a concentration of 500 μmol/L, and the expression of native and phosphorylated ERK and Pyk2 was examined by western blotting. EPA significantly suppressed ERK phosphorylation at 1 and 2 h, although there was no difference relative to the control group after 3 h (Fig. 7). A similar significant inhibitory effect was observed on Pyk2 phosphorylation at 2 and 4 h (Fig. 8). Treatment with the ROS inhibitor NAC (5 mM) alone did not affect Pyk2 phosphorylation, while NAC slightly enhanced the inhibitory effect of EPA on Pyk2 phosphorylation (Fig. 9).

## Discussion

The anti-cancer effects of n-3 PUFAs in vitro and in vivo are well known [13, 14, 18, 26–31], and these effects have been observed in PC cells. Our previous study showed that the n-3 PUFAs EPA and DHA inhibit PC3 cell proliferation, migration, and invasion [18]. Several reports have indicated that n-3 PUFAs-induced apoptosis or oxidative stress suppresses the growth of various types of cancer cells, including PC cells [32, 33]. Activation of NRTKs may be related to PC cell growth, and clinical trials of therapeutics that target NRTK are currently underway [34, 35]. Other mechanisms of action have been proposed for the anti-cancer effects of n-3 PUFAs [36, 37]. In this study, we addressed this question using phosphokinase and apoptosis antibody arrays and examined the contribution of ROS to the anti-cancer effect of EPA on PC3 cells.

For the antibody array analysis, the cell cycle of PC3 cells was synchronized by culturing in serum-free medium for 24 h. PC3 cells are relatively resistant to the anti-cancer effects of EPA. We treated cells with a moderately high concentration of EPA that was nonetheless within the physiological range (500 μM). PC3 cell proliferation did not change over 24 h in the control group, which is consistent with the notion that the rates of proliferation and death are equivalent in PC3 cells grown under conditions of moderate nutrient starvation. In contrast, the number of cells in the EPA-treated group decreased over time, with significant differences relative to the control group observed from 3 h to 24 h.

The results of the apoptosis array showed that EPA increased the expression of the pro-apoptotic proteins TRAIL-R1, caspase 8, and p53, and decreased that of the anti-apoptotic protein Bcl-2. However, EPA also increased the levels of the anti-apoptotic proteins SMAC and Survivin while having no effect on caspase 3. We speculated that ROS induced cell death via a pathway independent of caspase-induced apoptosis. To test this hypothesis, we evaluated the effect of the ROS inhibitor NAC on EPA-treated cells. First, we measured ROS generation at two time points, 2 and 24 h. In these experiments, we observed relevant (not significant) ROS generation induced by EPA only at 2 h, and NAC reduced the ROS level to the control (data not shown). We observed that the addition of NAC abrogated the anti-cancer effects of EPA (Fig. 6), suggesting that ROS induced by EPA suppressed PC3 cell proliferation. This is supported by previous reports showing that the anti-cancer effects of n-3 PUFAs are mediated by ROS in liver cancer and PC cells [23, 24].

We also investigated the contribution of cell proliferation pathways to the anti-cancer effects of EPA using a phosphokinase antibody array. The results showed that Pyk2, eNOS, c-Jun, and RSK1/2/3 were downregulated by over 20% by EPA treatment as compared to the control group. Western blot analysis revealed that ERK and Pyk2 phosphorylation were inhibited by EPA (Figs. 7 and 8). Suppressing ROS production with NAC did not affect the inhibitory effects of EPA on Pyk2 phosphorylation, suggesting that the suppression of Pyk2 phosphorylation by EPA does not involve modulation of ROS. Pyk2 is a member of the focal adhesion kinase (FAK) family of NRTK and plays important roles in cell survival, proliferation, and migration [38–41]. In our experiments, FAK phosphorylation was also suppressed by EPA treatment (Table 2). FAK is localized at the site of cell adhesion with the extracellular matrix along with integrins, whereas Pyk2 associates with integrin within the cell [42, 43]. Since several types of integrins are expressed in PC3 cells and interact with extracellular matrix, inhibition of activated FAK at these sites may prevent cell adhesion and migration [44]. The N-terminal domain of FAK has phosphorylation sites for the cell proliferation-related factors epidermal growth factor receptor, c-Met, and Src [45]. The antibody used in the antibody array detected phosphorylated tyrosine 402 of Pyk2, which is necessary for Src activation [40] and was slightly reduced in our experiment (Table 2). It was reported that Pyk2 activation induced smooth muscle contraction by activating Rho-associated kinase signaling [46], and FAK and Pyk2 show similar activities on this pathway in the regulation of cell migration [38, 47, 48]. Activation of both factors leads to abnormalities in cell morphology and movement associated with malignant transformation. Our results suggest that EPA negatively regulates the proliferation, migration, and invasion of PC3 cells via inhibition of Pyk2 and FAK [18].

Pyk2 is an NRTK that is activated by increased intracellular Ca^2+^ concentrations [49, 50], which are in turn increased by n-3 PUFAs and ROS [23, 51–53]. In our results, the n-3 PUFA EPA inhibited Pyk2 phosphorylation, whereas ROS inhibition by NAC did not affect the inhibitory effects of EPA, suggesting that EPA blocks Pyk2 activation independent of intracellular Ca^2+^ levels.

## Conclusions

**Fig. 10:**
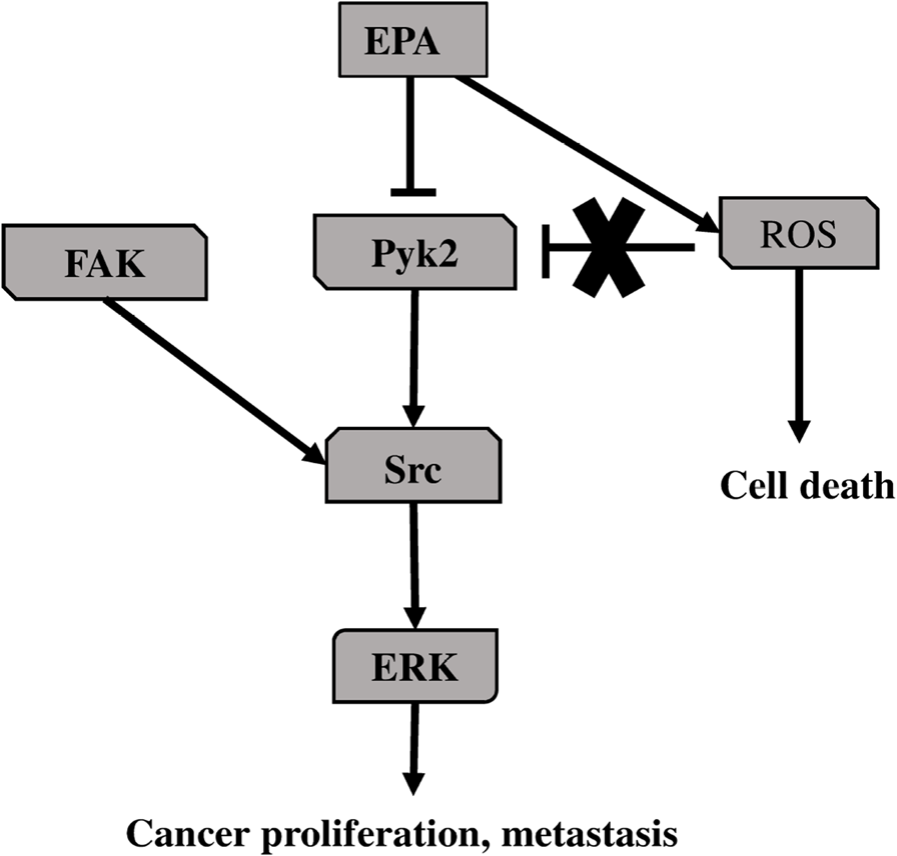
EPA-induce anti- cancer signaling in PC3 cells

Our results demonstrate that EPA induced cell death by stimulating ROS production in PC3 cells, and suppressed Pyk2 phosphorylation inducing inhibition of cell proliferation, migration, and invasion partly controlled by downstream Pyk2 signaling. Our findings were summarized in Fig. 10. These results provide a basis for the development of novel therapeutics for the treatment of PC.

## Data Availability

Available.

## Abbreviations

ADT: Androgen deprivation therapy
Bcl-2: B-cell lymphoma 2
CRPC: Castration-resistant prostate cancer
DHA: Docosahexaenoic acid
eNOS: Endothelial nitric oxide synthase
EPA: Eicosapentaenoic acid
ERK: Extracellular signal-regulated kinase
FAK: Focal adhesion kinase
n-3 PUFA: n-3 polyunsaturated fatty acid
NAC: N-acetyl cysteine
NRTK: Non-receptor tyrosine kinase
PBS: Phosphate-buffered saline
PC: Prostate cancer
Pyk2: Proline-rich tyrosine kinase-2
ROS: Reactive oxygen species
RSK1/2/3: Ribosomal S6 kinase 1/2/3
RTK: Receptor tyrosine kinase
SMAC: Second mitochondria-derived activators of caspase
sTNF-R2: Soluble tumor necrosis factor receptor 2
TRAIL-R1: Tumor necrosis factor-related apoptosis-inducing ligand and receptor 1
